# Sequencing error profiles of Illumina sequencing instruments

**DOI:** 10.1093/nargab/lqab019

**Published:** 2021-03-27

**Authors:** Nicholas Stoler, Anton Nekrutenko

**Affiliations:** Graduate Program in Bioinformatics and Genomics, The Huck Institutes for Life Sciences, The Pennsylvania State University, University Park, PA 16802, USA; Department of Biochemistry and Molecular Biology, The Pennsylvania State University, University Park, PA 16802, USA

## Abstract

Sequencing technology has achieved great advances in the past decade. Studies have previously shown the quality of specific instruments in controlled conditions. Here, we developed a method able to retroactively determine the error rate of most public sequencing datasets. To do this, we utilized the overlaps between reads that are a feature of many sequencing libraries. With this method, we surveyed 1943 different datasets from seven different sequencing instruments produced by Illumina. We show that among public datasets, the more expensive platforms like HiSeq and NovaSeq have a lower error rate and less variation. But we also discovered that there is great variation within each platform, with the accuracy of a sequencing experiment depending greatly on the experimenter. We show the importance of sequence context, especially the phenomenon where preceding bases bias the following bases toward the same identity. We also show the difference in patterns of sequence bias between instruments. Contrary to expectations based on the underlying chemistry, HiSeq X Ten and NovaSeq 6000 share notable exceptions to the preceding-base bias. Our results demonstrate the importance of the specific circumstances of every sequencing experiment, and the importance of evaluating the quality of each one.

## INTRODUCTION

Assessing the accuracy of next-generation sequencing has been the focus of much study since these techniques emerged. In 2011, studies on the Illumina Genome Analyzer (GA) and GA IIx discovered an association between errors and certain sequence motifs leading up to the error site (1,2). One of the studies also produced a profile of substitution biases, including a strong preference for T-to-G substitutions (2). This study made use of a phenomenon where mates in paired-end sequencing experiments ‘overlap’. In this situation, the ends of the two reads cover the same portion of their source fragment. This allows sequencing errors in one read to be revealed by the other.

In the past few years, many new sequencing instruments have been introduced. For instance, Illumina has introduced the HiSeq X Ten, with patterned flowcells, NextSeq 500, with 2-dye chemistry and NovaSeq 6000, combining both in an industrial-scale platform (3). While the basic reversible chain-terminator principle remains unchanged, these are significant modifications which could be expected to introduce their own biases. For instance, labeling nucleotides with only two fluorophores means that guanine is detected by the absence of signal (3). Some have reported that this results in overcalling of G’s when artifacts cause signal dropout (4). On the other hand, a controlled study compared HiSeq 2500 and NovaSeq 6000 and indicated a lower error rate in the NovaSeq (5). Evidently, these new technologies beg examination to determine their effects on sequencing errors.

Comparing the error rates of sequencing platforms has been a focus of research since sequencing began. Every new platform has its advantages and disadvantages, with its error rate being one of the most important factors. Typically the error rate is assessed by comparison of results across different platforms with multiple replicates (6). This is the gold standard for showing how the different technologies operate in the same hands. These studies are useful when one is deciding on an instrument to use. But different groups see different outcomes with the same technology. Even within the same group, there is often variation from experiment to experiment (7). And there may be a difference between error rates observed in an ideal scenario versus typical use ‘in the wild’. So, knowing the extent of this variation is important for consumers of sequencing data produced by others. Even researchers choosing technologies for their own data may find it useful to know how much their mileage may vary.

But measuring error is a theoretically difficult task. Some have taken a simple approach, aligning reads to a reference and calling variants as errors (6). But real variants will then be misclassified as errors as well. Instead, one could first perform variant calling, assuming the majority allele at any position is correct and any minor alleles are errors. This will work well for samples that are known to be highly homogeneous, but otherwise there may be true minor alleles which would be mistaken for errors (8). This can be the case for samples of microorganisms, viruses, cancers or organelles. It is difficult to automatically ascertain how homogeneous a sample is, making it a hurdle for an automated survey. Also, at sites with low numbers of reads, it is possible that the error base randomly occurs more often than the true sample base, causing artifacts in error detection. Another issue with both of these approaches is that they detect errors from more than just sequencing. Library preparation steps like polymerase chain reaction (PCR) can also introduce errors. And different preparation techniques can introduce different numbers and types of errors. Both of the error detection methods above will identify both library preparation errors and sequencing errors combined.

**Figure 1.**
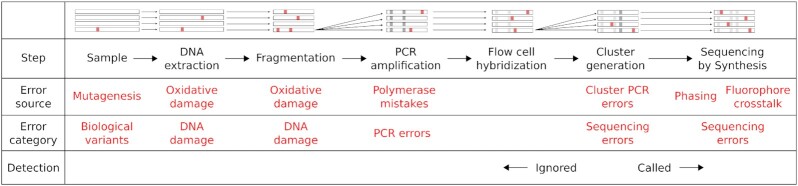
The steps involved in sequencing a biological sample, and where polymorphisms can arise. This shows a typical procedure to extract DNA from a sample, prepare a sequencing library and sequence it. Different types of variants are labeled at the point where they can be introduced. The bottom row indicates which variants are ignored by overlap analysis and which are detected and considered ‘sequencing errors’. At the top is a visualization of what happens to the DNA in the sample at each step. Arrows indicate the lineage of DNA molecules, either where they are the same actual molecule (horizontal arrows) or copies of the same ancestor (angled arrows). The visualization focuses on the ancestor molecules of one Illumina flow cell cluster (right). So at PCR amplification and cluster generation, we ‘zoom in’ to focus on copies of the single molecule which is an ancestor. Red spots indicate variants which have been introduced, and gray spots indicate where a variant from a previous step becomes fixed in the molecules being shown. These gray variants are no longer polymorphic among the ancestors of that single, final cluster. These fixed variants are ignored by our error detection method.

In a paired-end experiment, when a fragment is smaller than the length of both reads combined, the ends of the reads will overlap. This means that, in this overlapping region, the same DNA fragment is assayed twice. Both reads share this same exact molecule as an ancestor, and the only source of errors in-between is from the sequencing instrument. Any PCR errors, cloning polymorphisms, DNA damage or other library preparation errors that have occurred have already been introduced into the fragment (Figure 1), and will not produce a difference between the two reads (6). This provides a powerful method of assaying the sequencing error introduced by an instrument in any paired-end dataset with sufficient overlap.

Armed with a method that can be retroactively applied to a large portion of existing datasets, we can then perform a large-scale survey of real-world sequencing experiments. The Sequence Read Archive (SRA) hosts the largest public database of next-generation sequencing data (9). The SRA provides metadata which can allow automatically filtering for qualifying datasets and categorizing them by sequencing platform. In order to enforce uniformity, we decided to focus only on one organism: *Escherichia coli*. We chose *E. coli* because of its relatively compact genome, making sequence alignment simpler. It is one of the most studied prokaryotes, with a large number of publicly available datasets. Also, in order to focus on new Illumina technologies, we scoped our survey to only this manufacturer.

## MATERIALS AND METHODS

### Obtaining SRA datasets

We selected *E. coli* datasets from the SRA using the Entrez Direct utilities from NCBI (10). Using the query ‘Escherichia coli’[Organism], we fetched the metadata for all 186 022 matching runs as of 31 August 2020. A total of 179 306 of these were by Illumina instruments, with 75 118 MiSeq, 36 034 HiSeq 2500 and 1375 NovaSeq 6000. A full breakdown is given in Supplementary Table S1. We then filtered out single-ended and non-Illumina datasets, ordered the list to prefer a diversity of sequencing platforms and submitting groups, and prioritized runs that were the most likely to have the most read overlap. We then downloaded the FASTQ files using the SRA toolkit (version 2.10.0) or EBI’s FTP server.

### Determining the best reference

In order to determine the best reference sequence for read alignment, we performed a ‘meta-alignment’ where we combined all complete *E. coli* genomes into one reference and aligned the sample reads to it. We used the NCBI Genome database to gather a list of complete genomes. Specifically, we downloaded the table available at ftp.ncbi.nlm.nih.gov/genomes/refseq/bacteria/Escherichia_coli/assembly_summary.txt and selected all assemblies with an assembly level of ‘Complete Genome’. This was 1292 assemblies as of 31 August 2020. We downloaded the FASTA files from the ftp_path in that table and concatenated them into a single meta-reference. For each sample, we aligned its reads to the meta-reference with BWA-MEM (11) (with the -M flag; version 0.7.17-r1188). Then we read the alignments with samtools (12) to count how many alignments were made to each reference. We only counted primary alignments (SAM flag 256) which were mapped (flag 4), passed instrument QC (flag 512), were not PCR/optical duplicates (flag 1024), and not supplementary (flag 2048). Then we chose the reference with the most alignments, excluding references smaller than 2 Mb.

### Detecting overlap errors

Detecting errors began by aligning the reads of each run to the chosen reference. Alignment was performed with bwa mem -M, as in the previous section. Reads were removed if they did not belong to a pair where both reads were mapped (had SAM flags 1 and 2, and not 4 or 8). They were also removed if they were secondary alignments (flag 256), failed instrument QC (flag 512), were PCR/optical duplicates (flag 1024) or were supplementary alignments (flag 2048). Then, errors were detected by pairing read bases by their reference coordinate, reporting mismatching bases as errors.

### Calculating error rates

We calculated the error rate of each sample by dividing the number of detected errors by the amount of overlap between read pairs. The calculation excluded errors where one base was N. We also broke down the error rate for each sample by regions of the reads. For each error, we determined where in both reads it occurred. Then we divided each read into bins 1/10th of the read length long. We defined the bin of the error to be the bin of the read length it occurred in. If it was in different bins in the two reads, we took the greatest of the two (the bin furthest toward the 3′-end of the read). This is because the main purpose of binning is to reduce the effect of errors increasing toward the end of reads. If an error appeared in bin 2 of one read, and bin 10 of its mate, it is more likely to be from a sequencing error in the latter read, occurring due to how close to the end of the read it is. After the binning process, we calculated the error rate separately for each bin. For each sample, we required at least 2.5 million overlapping bases in a bin to calculate a valid error rate for it.

### Correlating error rates with platform and lab

We selected the samples with valid error rates calculated in the previous section using the rates as our dependent variable. Our independent variables were derived from the ‘model’, ‘center’, ‘lab’ and ‘contact’ metadata fields. The latter three were combined and each combination was deemed a separate ‘group’. In order to reduce the number of categories, we combined all groups which appeared less than four times into an ‘other’ group. We then performed ordinary least squares regression with the model and group as the independent variables and the error rate as the response variable. The regression was performed by the statsmodels.formula.api.ols function from the statsmodels Python package.

### Tabulating base frequencies in error sequence contexts

For every genomic location where we detected an error, we extracted the 20 bp of genomic sequence centered on the error site. For each substitution in each platform, we counted the total count of each base at each distance from the error. We determined the substitution by first examining all the read bases at the error site. We chose the most common base as the most likely major allele in the sample. Then, for each error, we assumed the base that did not match the major allele was likely the erroneous base. If neither read base matched the major allele, we did not call the substitution or include it in the analysis. Once all the substitutions were called, we chose the most common one at that site.

### Calculating trimer frequencies

For every genomic location with a detected error, we examined the three genomic bases leading up to, and including the error site. For every platform, we counted how many trimers of each type were present in the set of unique error loci. We converted the counts to frequencies by dividing by the total number of trimers. We then normalized the frequencies by the prevalence of each trimer in the genome. We chose the most common reference sequence in our samples, NZ_CP044311.1 from strain RM13752. We counted the number of each trimer in that sequence, then converted to frequencies. We then divided each trimer's error frequency by its genomic frequency.

### Counting post-homopolymer errors

A particular error pattern has been observed in Illumina in regions with homopolymer runs. After a homopolymer of a particular base, the base immediately following the homopolymer will often be subject to a substitution where the error base is the same as the homopolymer base (1). Here, we refer to this error type as a ‘post-homopolymer error’.

We examined the sequence context surrounding every detected error, counting errors which matched the post-homopolymer pattern. This included every error where the substituted base matched the previous genomic base. We assumed the substituted base was the base that did not match the genomic (reference) base at that site. In the raw data, we included the identity of the preceding/error base and how many times it was repeated. For Figure 6, we normalized the error counts by the frequency of homopolymers in the genome. To do this, we analyzed the same genome as for the trimers, NZ_CP044311.1, counting the number of homopolymers of each base type and length. We then determined the number of post-homopolymer errors of each type we would expect at random. To do this, we first calculated the per-base frequency of each homopolymer type in the genome: the number of homopolymers of that base and length divided by the length of the genome. To get the expected number of random post-homopolymer errors, we multiplied this frequency by the total number of errors detected, and divided by four.

## RESULTS AND DISCUSSION

### Distribution of error rates for each platform

**Figure 2.**
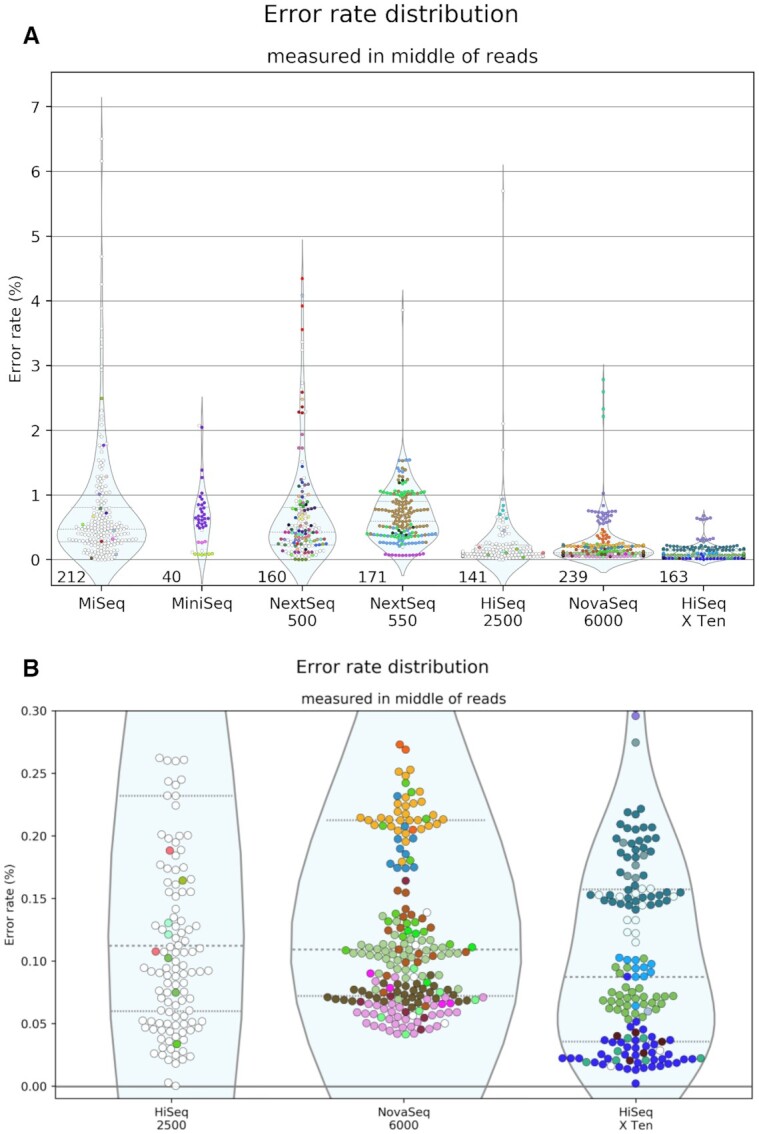
Error rates calculated from the overlap between read pairs. Errors were counted from the regions of reads 50–60% of the way through their lengths. Each SRA run is shown as one point. Only runs with sufficient overlap are shown; each must have a total of at least 2.5 Mb in the 50–60% bin. The number of runs shown is displayed at the bottom of each distribution. A different color was given to each group with more than three samples in the survey. Groups with three or fewer are colored white. Groups are defined by the combination of the center, lab and contact metadata fields. Panel (**A**) displays all instruments in the survey with more than 10 passing runs. Panel (**B**) is a zoom on the low-error instruments, showing only runs with an error rate <0.3%.

Figure 2 shows the range of error rates in samples from different platforms. For each read pair, errors were only chosen from the region between 50 and 60% of the full read length. This controls for the variation in overlap between samples. Error rate is highly correlated with the sequencing cycle, rising toward the end of each read. In samples with smaller overlaps, the detected errors will tend to be later in the reads than in samples with larger overlaps. To reduce this undesired influence, we selected errors from similar regions in the reads.

The median error rate of each platform is shown in Table 1. These vary from 0.087% in HiSeq X Ten to 0.613% in MiniSeq. These figures are comparable to those determined in more controlled settings (8,13). Perhaps even more striking is the variation within each platform. The error rates vary far more between samples than between platforms. Previous studies have shown small-scale indications of this phenomenon (5,7). Ma *et al.* shows that some portion of this variation may come from oxidative damage introduced by differential sample handling (5).

**Table 1.** Summary statistics for the observed error rates of samples from each sequencing platform

		Error rate (%)	
Platform	Number of samples	Median	Standard deviation
MiSeq	212	0.473	0.938
MiniSeq	40	0.613	0.459
NextSeq 500	160	0.429	0.827
NextSeq 550	171	0.593	0.435
HiSeq 2500	141	0.112	0.544
NovaSeq 6000	239	0.109	0.350
HiSeq X Ten	163	0.087	0.126

The samples and error rates summarized are the same shown in Figure 2.

While the error rates may not be significantly different, their variation does depend greatly on the platform. The highest standard deviation is in MiSeq, at 0.938 percentage points. The lowest is in HiSeq X Ten, at 0.126. There seem to be two categories of platforms—one with higher variation and error rates, and one with lower variation and error rates. The instruments in the latter category are HiSeq 2500, HiSeq X Ten and NovaSeq 6000—the most expensive machines. One explanation could be that users of these machines spend more time optimizing their runs, since a low-quality run would be a much more expensive loss.

The HiSeq X Ten is easily the most consistent platform. Since this instrument is such an expensive installation, we asked whether the consistency is due to the datasets being dominated by one lab group. So we colored Figure 2 by lab group, which shows that there are several groups which are represented much more than others, and the error rates do tend to be consistent within each group. A full list of lab groups is available in Supplementary Table S2. However, there is substantial diversity overall. Eleven different groups contributed to our HiSeq X Ten total, with nine contributing at least five samples. Another notable characteristic is the bimodal distribution of NovaSeq 6000 samples. This distribution is due to a cluster of 23 NovaSeq runs from one group clustered around 0.683%, rather than the 0.106% of the rest of the NovaSeq samples. While the NovaSeq median is technically lower than that of the HiSeq 2500, their error rates are very similar, in contrast to the marked difference evident in Supplementary Figure S4 of (5). And in contrast to early reports that HiSeq X Ten had higher error rates than older HiSeq instruments (14), our survey shows the error rate of public HiSeq X Ten datasets is even lower than HiSeq 2500, and more consistent.

In order to investigate how much the error rate of each sequencing run depends on the platform versus the group producing it, we performed linear regression on all the datasets. Supplementary Figure S1 shows that the coefficients for research groups are on a similar or greater scale than that of the sequencing instruments. On the other hand, Supplementary Figure S2 shows that just as many platforms as groups are significantly correlated with error rate, after Bonferroni correction. Figure 2 appears to show clear group-specific patterns, and this test was able to show significant correlation in three of the groups. On the other hand, three sequencing platforms were also shown to correlate with accuracy. So this test does support the idea that despite great intra-platform variation, accuracy still does depend on the sequencing platform.

### Base frequencies near error sites

**Figure 3.**
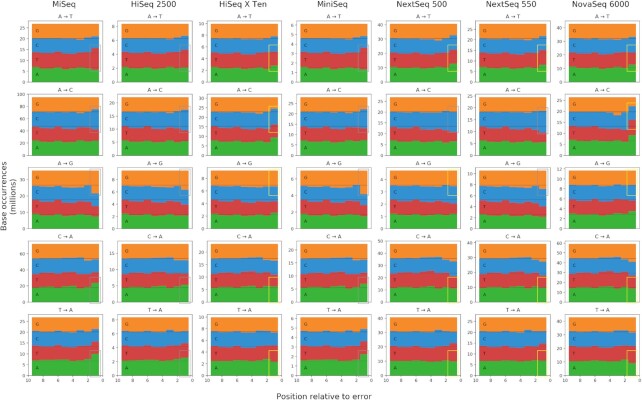
The count of each base in the genomic context surrounding each type of substitution. The *X*-axis represents the distance from the error base. The rightmost slot is the closest (adjacent to the error base), and the leftmost is the furthest (nine bases from the error). The *Y*-axis is the count of how many of each base was observed at each distance from a substitution of that type. Boxes highlight the count of the error base adjacent to the error. Gray boxes surround counts which are over-represented, as expected. Yellow boxes surround counts which do not seem to follow this pattern.

Studies of errors in Illumina sequencing have consistently shown the importance of sequence context. In order to investigate whether there are platform-specific differences, we examined the genomic context surrounding each error we detected. Figure 3 shows the frequency of each base at each position relative to each error. We further divided each platform by the type of substitution, focusing in this case on errors that involved adenine (A).

Most platforms followed the common Illumina bias toward substituting a base of the same type as the one preceding the error. But this phenomenon was inconsistent. Neither HiSeq X Ten nor NovaSeq 6000 showed much evidence of this trend when looking just at the cumulative base counts. The same was true for both NextSeq platforms, but only in the case of A→T, C→A and T→A substitutions. And in all of the substitutions away from A where this phenomenon was missing, A was over-represented instead.

Notably, these patterns do not show clear signatures of errors due to the pattern of fluorophore dyes in the respective instruments. MiSeq and HiSeq use traditional four-color imaging, where the wavelengths for C and A overlap and T and G overlap (15). But Figure 3 does not show the C/A and T/G associations one might expect from this overlap. And MiniSeq, NextSeq and NovaSeq use two-color imaging, where A shares a wavelength with both C and T, and G is unlabeled. There have been reports of this resulting in overcalling of G’s, leading to stretches of polyG’s (4). PolyG’s would result in a greater over-representation of G’s leading up to a G substitution. But this is not clearly observed in the plots. MiniSeq may be an exception, but the effect is only seen in the immediately adjacent base, not any others. Another expected error would be C→A and T→A substitutions when preceded by T or C, respectively. This could occur when phasing causes the red from a preceding C to mix with the green of a T, or vice versa. This mixed red/green signal could be misread as an A, which is normally a red/green mix. But no platform seemed to show an over-representation of T adjacent to C→A substitutions or C next to T→A ones.

### Frequency of trimer motifs at error sites

**Figure 4.**
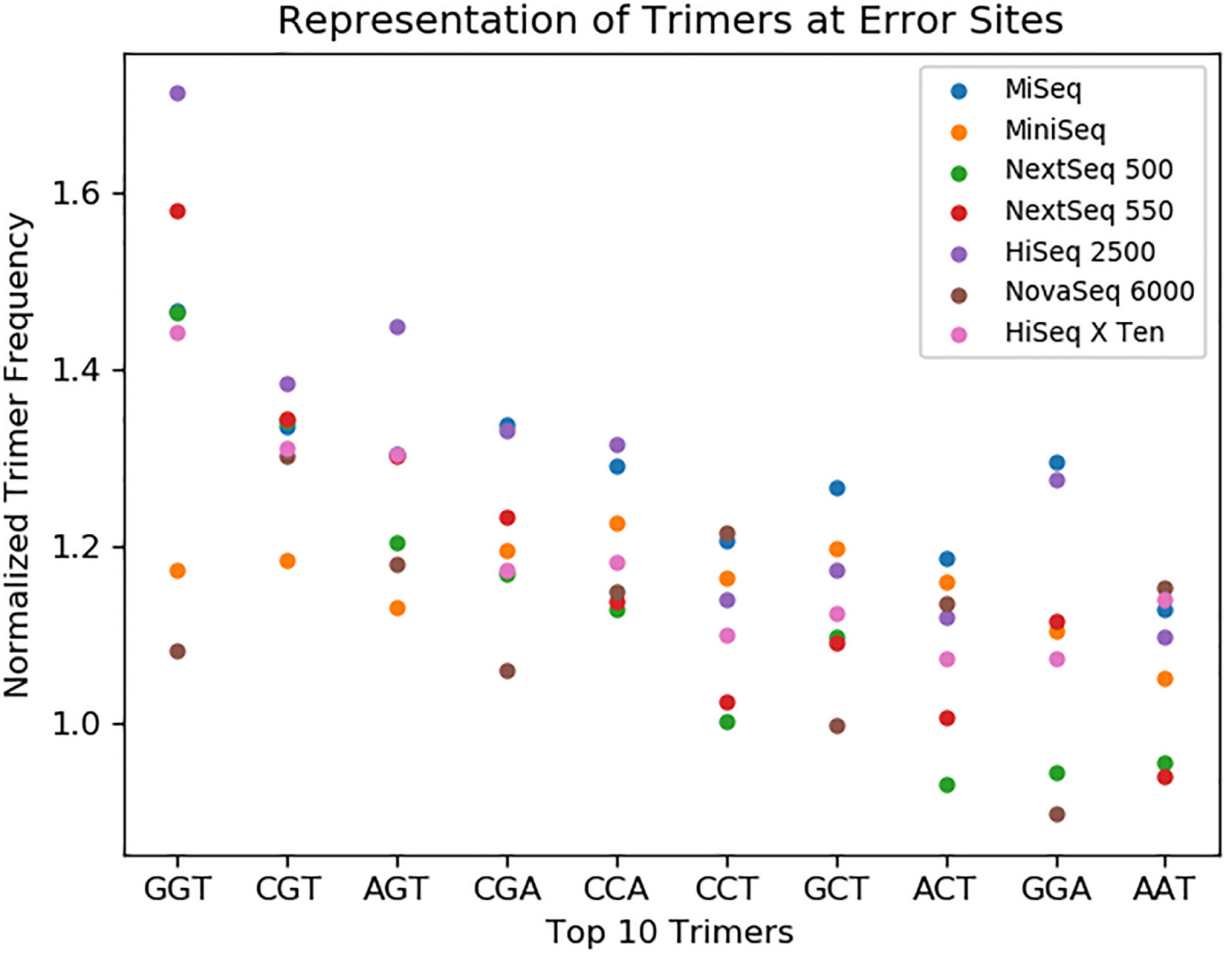
The frequency of trimers in the sequence context near errors. Each trimer is the three reference bases leading up to, and including, an error site. As described in the ‘Materials and Methods’ section, we counted the occurrences of each trimer, then normalized by the abundance of the corresponding trimer in the genome. A normalized frequency of 1 would mean the trimer is associated with errors exactly as much as in the null hypothesis where errors are randomly distributed. Trimers are presented in order of their median frequency among all platforms.

Certain sequence motifs are known to be especially error-prone under Illumina sequencing. We investigated the motifs associated with our errors to discover if there were platform-dependent differences. We checked the three bases leading up to, and including the error base at every error site. Figure 4 shows the frequency of each trimer at our error sites, normalized by the expected frequency if there were no correlation. The top 10 trimers in the entire survey are shown.

The most error-associated trimer in our dataset is GGT, a motif which has also been seen in previous studies (2,16). Most instruments show a similar trend, but there is wide variation. GGT is far more over-represented in HiSeq 2500 errors than any other platform. NovaSeq 6000 is at the low end of several of the top trimers, indicating it is less influenced by these motifs. In contrast, HiSeq 2500 and MiSeq seem to show the most motif-dependent errors.

### Differences in error rate types

A common error mode in Illumina platforms occurs near homopolymers. After a repeat of the same base multiple times, Illumina reads will often substitute the first base after the homopolymer with the homopolymer base. This can occur due to phasing—lagging molecules will still be incorporating homopolymer bases while the instrument is reading the post-homopolymer base (17,18).

**Figure 5.**
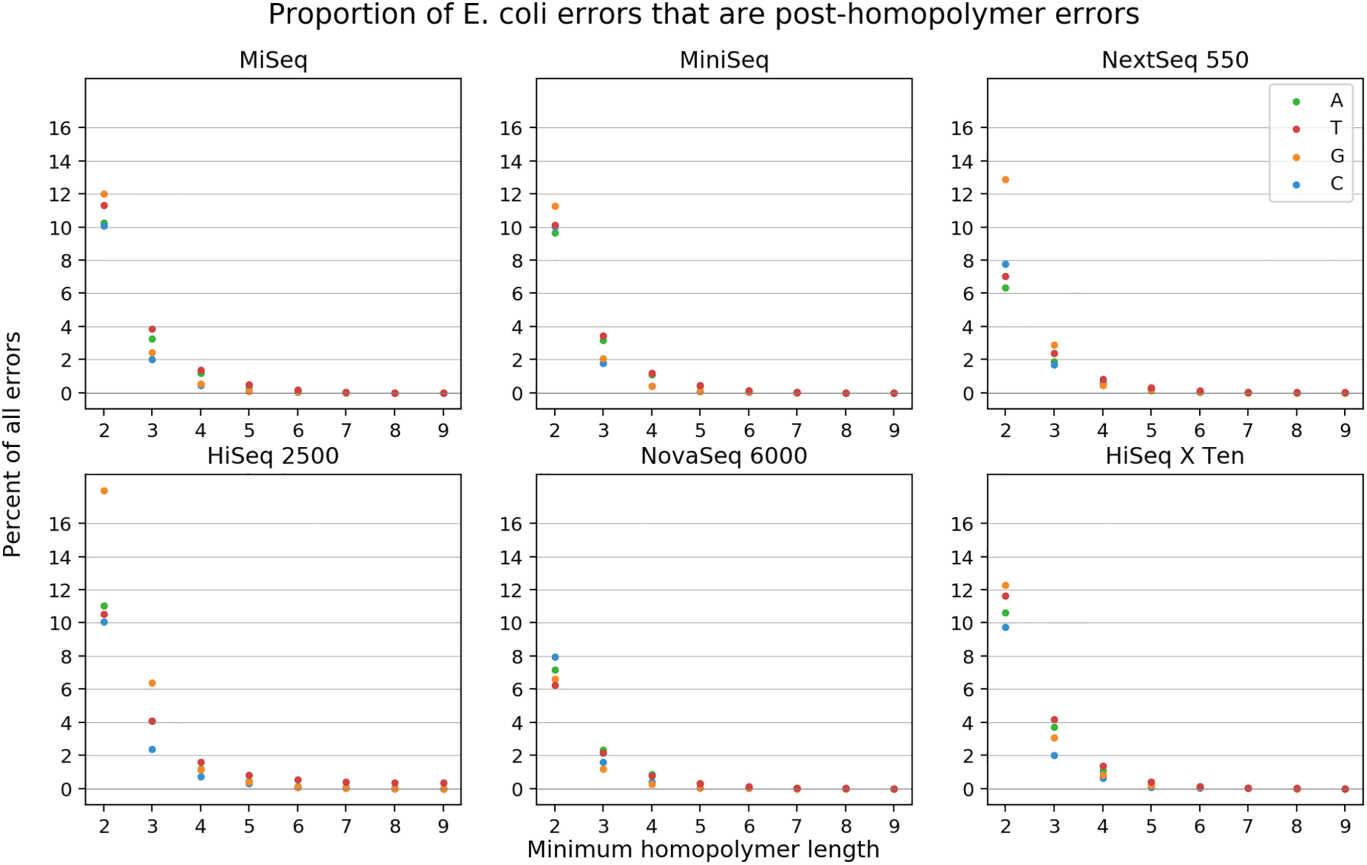
Frequency of post-homopolymer errors. For each platform, we found all errors where the error base is identical to the preceding genomic base. If the preceding genomic base is part of a single-base repeat (homopolymer), we might call the error a post-homopolymer error, depending on the length of the repeat. This figure shows what percent of all errors are post-homopolymer errors, depending on the threshold one uses for the definition of a post-homopolymer error. Numbers are broken out by error/homopolymer base. For example, if one decides that any error preceded by a run of at least three bases of the same identity qualifies as a post-homopolymer error, then about 2% of all C substitutions in MiniSeq are post-homopolymer errors.

In our survey, we observed that this type of error is common. If we define a homopolymer as any run of three or more of the same base, this error constitutes between 0.7 and 5.3% of all errors, depending on the base and platform. Figure 5 shows how common this error is, depending on how one defines a homopolymer and which base the homopolymer is composed of.

**Figure 6.**
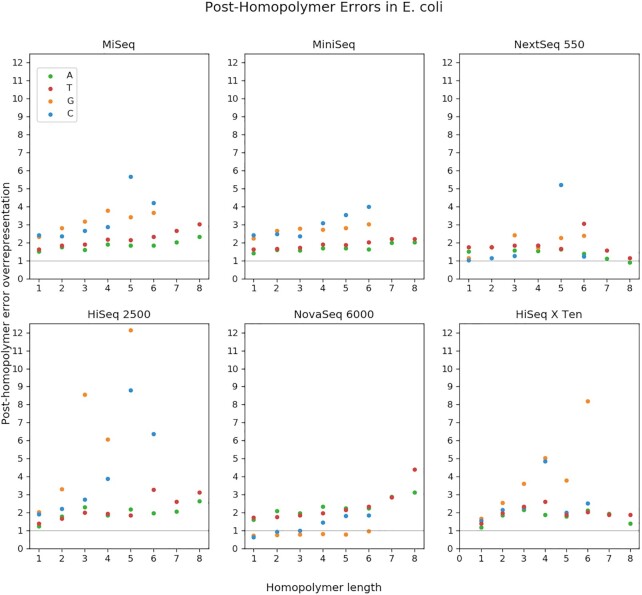
Relative frequencies of post-homopolymer errors in *Escherichia coli*, by length. Each post-homopolymer error was categorized by its error base and the length of the preceding homopolymer. Each total was divided by the expected number of errors of that type in the null hypothesis where errors are randomly distributed. An ‘over-representation’ of 1 is what would be expected in the null hypothesis, while 2 would be twice as abundant.

Figure 6 shows the rate of these errors relative to the neutral expectation if there were no correlation between errors and homopolymers. Each point is the number of homopolymer errors divided by the number of homopolymers of that type in the *E. coli* genome. So in MiniSeq, G substitutions follow G 3mers about three times more often than if substitutions were distributed randomly. As expected, across platforms, even the normalized rates increase with homopolymer length. Another pattern that is common across platforms is that G/C homopolymers produce errors at a higher rate than A/T homopolymers. Instruments which do not fully follow this pattern are NextSeq 550, HiSeq X Ten and NovaSeq 6000. Interestingly, in the latter case, the pattern seems to be inverted, with A/T more over-represented. Another peculiarity of NovaSeq 6000 is that G/C post-homopolymer errors rates are generally quite low, in many cases occurring less often than expected by random chance.

### Further examination

Here we developed a method which can be automatically applied to any paired-end sequencing dataset. We demonstrated its utility by applying it to a survey beyond the scale of manual annotation. We were able to show correlations between sequencing platforms, experimenters and error rates. But there may be other important factors explaining differences between samples. The cluster of NovaSeq 6000 samples with higher error rates, along with a cluster of six high-error HiSeq X Ten samples, were all produced by the Gene Expression Omnibus (GEO) group, which is a repository for expression data (19). This suggested that RNA-seq datasets may tend to have higher error rates. Luckily, information like this is captured in SRA metadata. Using that metadata, we reproduced Figure 2, colored by the LibraryStrategy field (Supplementary Figure S3). This reveals that all the GEO NovaSeq samples were in fact ChIP-seq. Only the HiSeq X Ten cluster was RNA-seq. Additionally, RNA-seq samples appeared at a wide distribution of error rates, showing no clear bias. However, the only ChIP-seq samples for these platforms were in that NovaSeq cluster. In the end, the commonality between these two clusters was the group (GEO), not the experiment type. This shows the power of the metadata to test hypotheses. And with links to other NCBI databases like BioProject and BioSample, there is even more metadata which could be automatically obtained about each sample and tested for correlations. For instance, a previous analysis of SRA metadata distinguished between published and unpublished datasets, as this may correlate with data quality (20). Unfortunately, the SRA metadata schema is far from complete, and there are some important features of a sequencing experiment which are not captured. For instance, the version of the Illumina reagent kit has been observed affecting the bias of sequencing data (6).

Our method of error detection has great advantages in its ease of automation, ability to be applied retroactively to a dataset, and its ability to identify errors coming from sequencing alone. But it can be made even less biased and automatable. In our survey, we relied on alignment to a reference sequence in order to find the overlaps in read pairs. In theory, the examination of overlaps does not require information from a reference at all. One could simply perform a two-way alignment of each pair of mates to each other. There are many pairwise alignment algorithms available, many of which will yield acceptable results even with default parameters. Some algorithms have trouble when there is minimal overlap between mates, but careful choice of algorithms and parameters can minimize this issue. By using pairwise alignment, we would simplify the workflow, eliminating the step that determines the best reference sequence. It would also eliminate possible biases from alignment artifacts. Additionally, it would remove the need to know anything about the subject of the sequencing experiment. This could allow surveying across organisms, so one could determine if that is a variable correlated with quality.

## DATA AVAILABILITY

The scripts used for the analysis are available on Github (https://github.com/makovalab-psu/overlaps).

Because of the lengthy compute time for the full analysis, we have also provided our intermediate data to assist in replicating our analysis (https://www.bx.psu.edu/nekrut_lab/overlap-survey/). With these files, one can repeat the analysis performed in the Jupyter Notebooks in the Github repository, generating all the results presented here. The sample directories should be placed in a ‘runs’ directory—the parent of runs will be the MAIN_DIR in Jupyter. The rest is explained in the Github README.md. The align.auto.bam files, which are the majority (1.8TB) of the data, are provided for transparency but are not necessary for the Jupyter analysis.

## Supplementary Material

lqab019_Supplemental_Files
