# Supplementary material for: Sequencing error profiles of Illumina sequencing instruments
Source: NAR Genom Bioinform. 2021 Mar 27;3(1):lqab019. doi: 10.1093/nargab/lqab019 (PMC8002175; doi:10.1093/nargab/lqab019)
Supplement: lqab019_Supplemental_Files [file lqab019_supplemental_files.zip › SupplementalFigures.pdf]

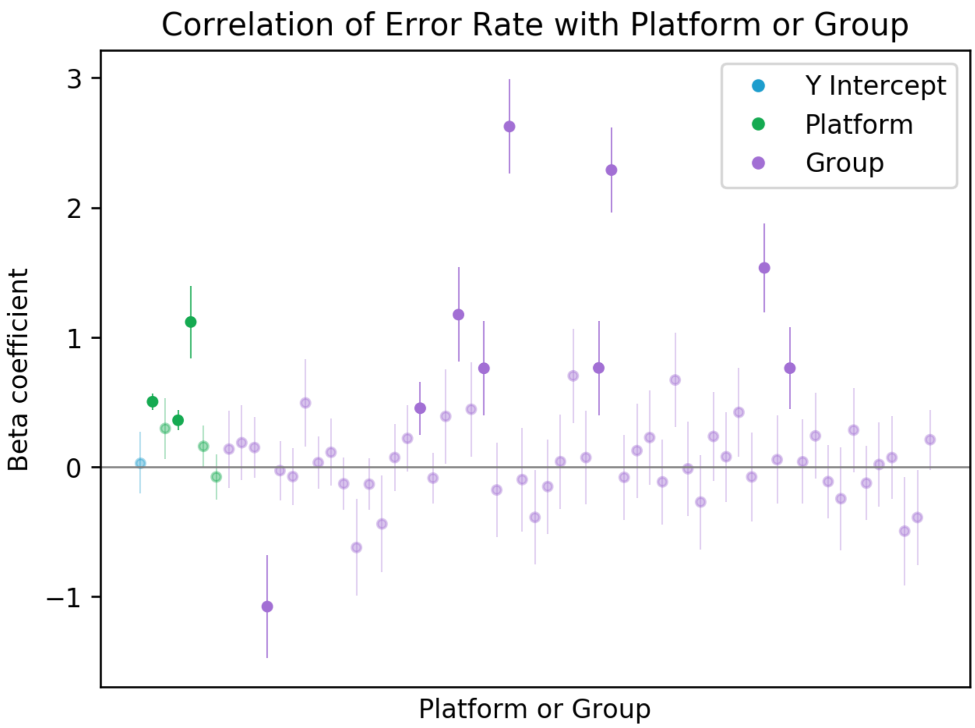

Figure S1: Regression coefficients for the correlation of platform and group with error rate. Each point represents a particular sequencing platform or dataset producer. Only groups which appear at least 4 times in the survey are included. Error bars represent standard error, and coefficients with a p-value ≥ 0.05 are shown lighter than significant ones. The platforms, from left to right, are the MiSeq, MiniSeq, NextSeq 500, NextSeq 550, NovaSeq 6000, and HiSeq X Ten.

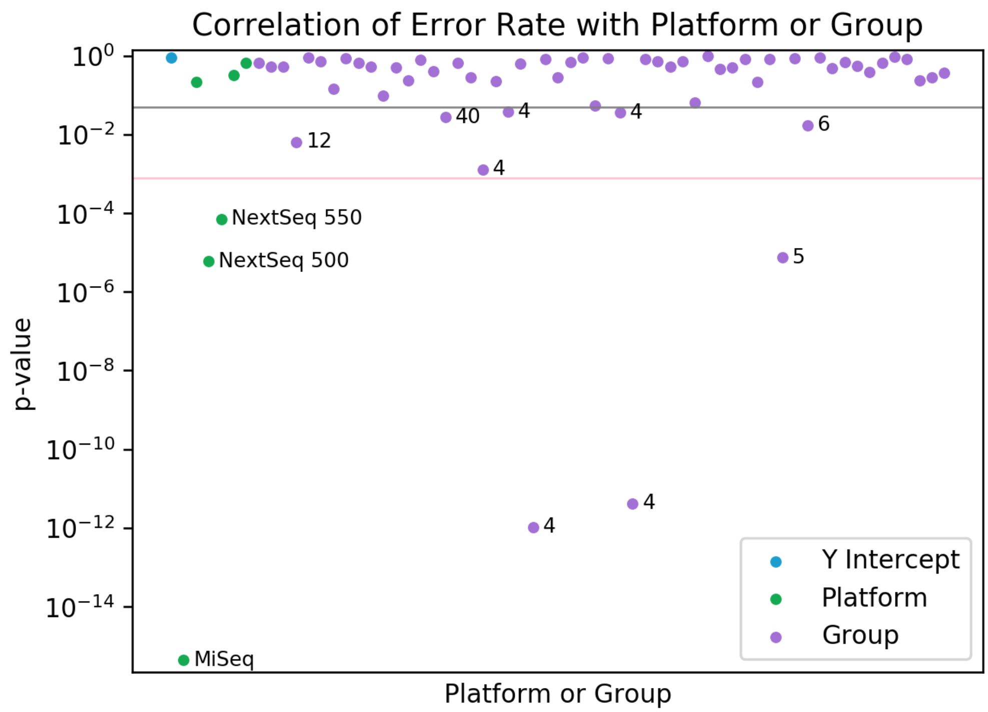

Figure S2: *P*-values of regression coefficients. The upper, gray horizontal line is a *p*-value of 0.05. The lower, red horizontal line is a Bonferroni-corrected *p*-value of 0.05/64 Significant platforms and groups are labeled. Group labels are the number of runs produced by the group that appear in the survey. The platforms are in the same order as in Figure S1.
