# Supplementary material for: Sequencing error profiles of Illumina sequencing instruments
Source: NAR Genom Bioinform. 2021 Mar 27;3(1):lqab019. doi: 10.1093/nargab/lqab019 (PMC8002175; doi:10.1093/nargab/lqab019)
Supplement: lqab019_Supplemental_Files [file lqab019_supplemental_files.zip › Supplementary file.docx]

# Supplementary Tables and Figures

[**Supplementary Tables**](https://docs.google.com/spreadsheets/d/1F9JQ99nOGy8Cvb4VJ1KSKOipi0JEMtGxi_r1LulHi4E/edit?usp=sharing)

**Table S1**: The distribution of SRA *E. coli* runs by sequencing instrument as of 31 August 2020.

**Table S2**: The submitter metadata for every unique group represented in this analysis. Groups were defined by the combination of the center, lab, and contact metadata fields. Included are groups which appear in the set of runs with a sufficient number of overlapping bases in the center bin, as defined in the methods. For brevity, some words are abbreviated, and long metadata values are truncated. The full values are available using the accession numbers provided from example runs.


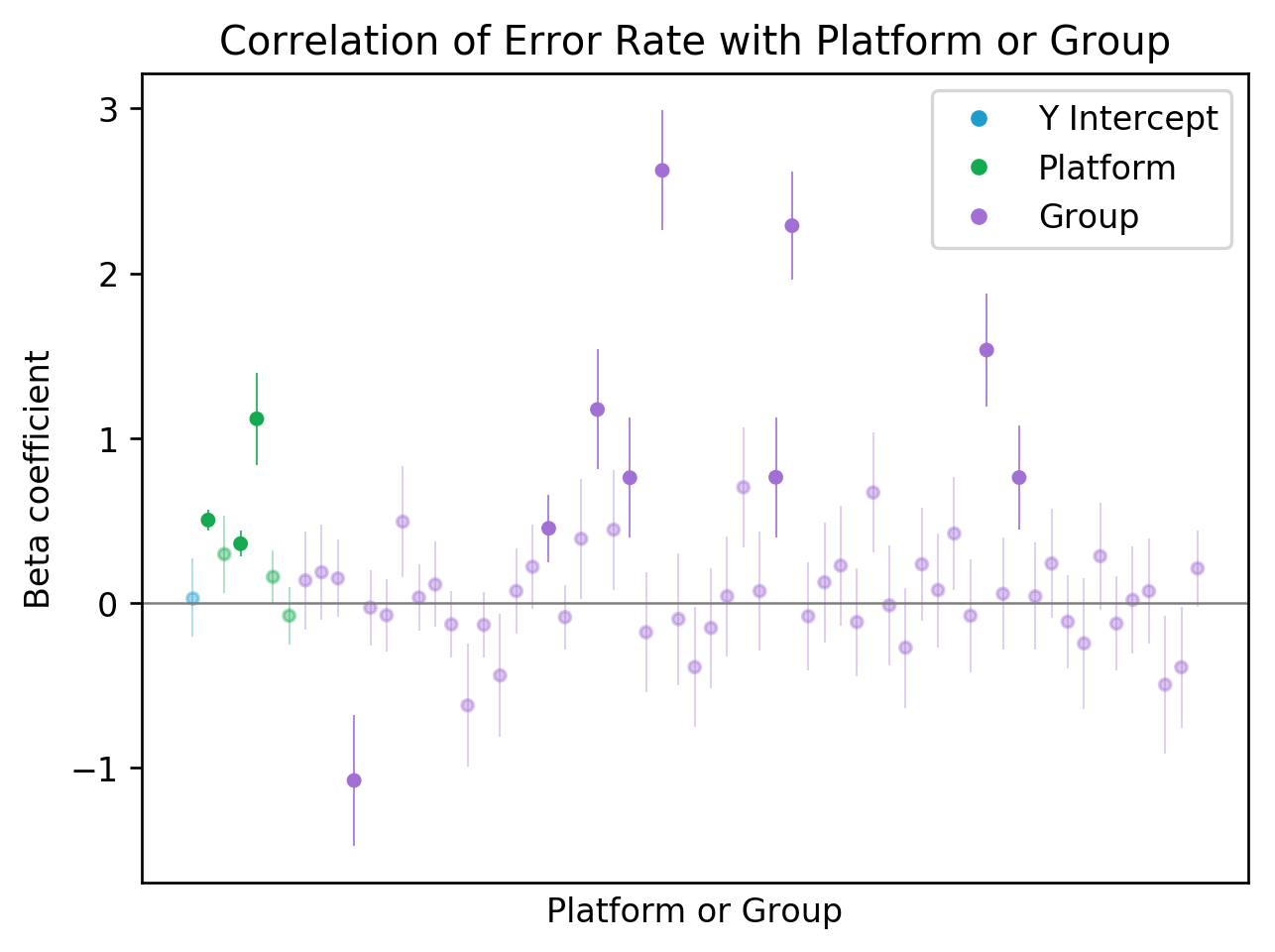


**Figure S1**: Regression coefficients for the correlation of platform and group with error rate. Each point represents a particular sequencing platform or dataset producer. Only groups which appear at least 4 times in the survey are included. Error bars represent standard error, and coefficients with a p-value ≥ 0.05 are shown lighter than significant ones. The platforms, from left to right, are the MiSeq, MiniSeq, NextSeq 500, NextSeq 550, NovaSeq 6000, and HiSeq X Ten.


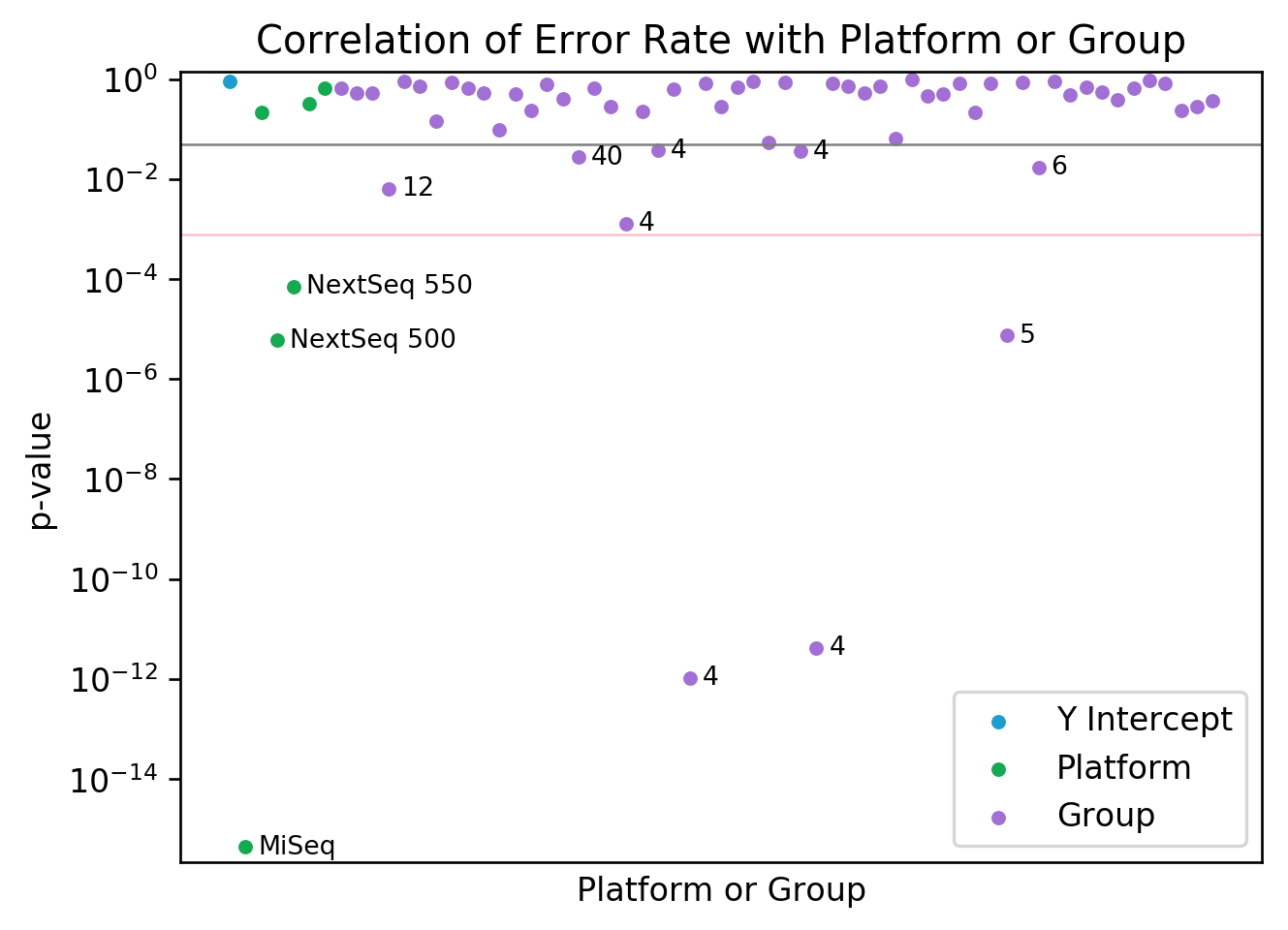


**Figure S2:** *P*-values of regression coefficients. The upper, gray horizontal line is a *p*-value of 0.05. The lower, red horizontal line is a Bonferroni-corrected *p-*value of 0.05/64 Significant platforms and groups are labeled. Group labels are the number of runs produced by the group that appear in the survey. The platforms are in the same order as in Figure S1.


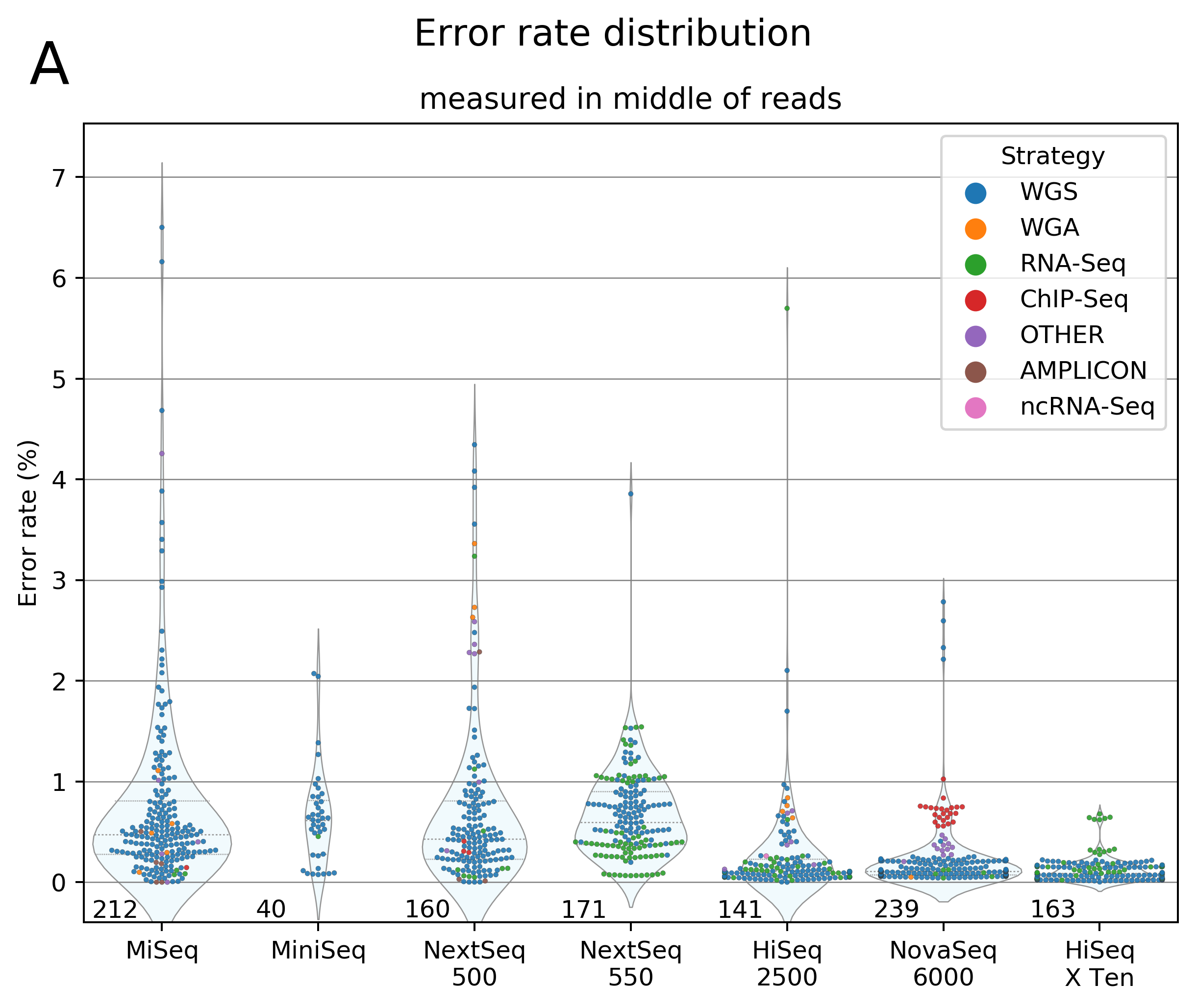


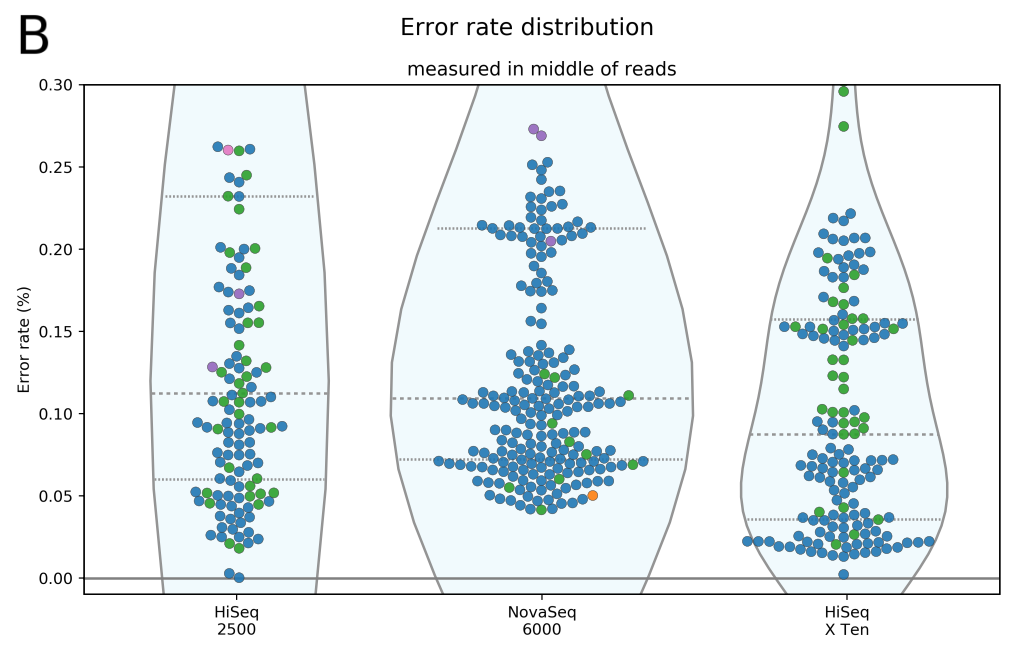


**Figure S3:** Error rates calculated from the overlap between read pairs, colored by library protocol. This is identical to Figure 2, but with the runs colored by the value of their LibraryStrategy SRA metadata field.

# Supplemental Methods

## Prioritizing SRA Runs

With 179,306 Illumina E. coli runs in the SRA, there were far too many to analyze them all. So we used the metadata to prioritize runs likely to have enough overlap to yield useful data. We did this by creating a queue of runs ordered by priority. The algorithm to create and use the queue was as follows. First we created a list of runs sorted by the predicted average read overlap (longest overlap first).^A^ Then, in order to avoid overrepresenting any particular lab group, we divided this list into sublists, one for each submitting lab group, preserving the ordering by overlap size. The lab group was identified by a tuple of (center, lab, contact) metadata values. We then created a new master queue by cycling through the lab group lists, picking one from each until they were all exhausted.^B^ Then the job scheduler would work through that queue in order, choosing a specified mix of Illumina instruments, according to the evolving totals of useful runs with sufficient overlap. The adjustments to the instrument mix were done to select our instruments of interest and correct for under-represented instruments.

^A^ The code for the sorting criteria can be found in the sort_key() function defined in cell 11 of [2020-09-01-ecoli-job-control.ipynb](https://github.com/makovalab-psu/overlaps/blob/master/jupyter/2020-09-01-ecoli-job-control.ipynb).

^B^ The code for dividing the list by group and cycling through them is in the divide_accessions_by_group() and get_diverse_runs() functions in cell 125 of [2020-09-01-ecoli-job-control.ipynb](https://github.com/makovalab-psu/overlaps/blob/master/jupyter/2020-09-01-ecoli-job-control.ipynb).
